# Supplementary figures and images for: Cell Wall–Based Machine Learning Models to Predict Plant Growth Using Onion Epidermis
Source: Int J Mol Sci. 2025 Mar 24;26(7):2946. doi: 10.3390/ijms26072946 (PMC11989001; doi:10.3390/ijms26072946)

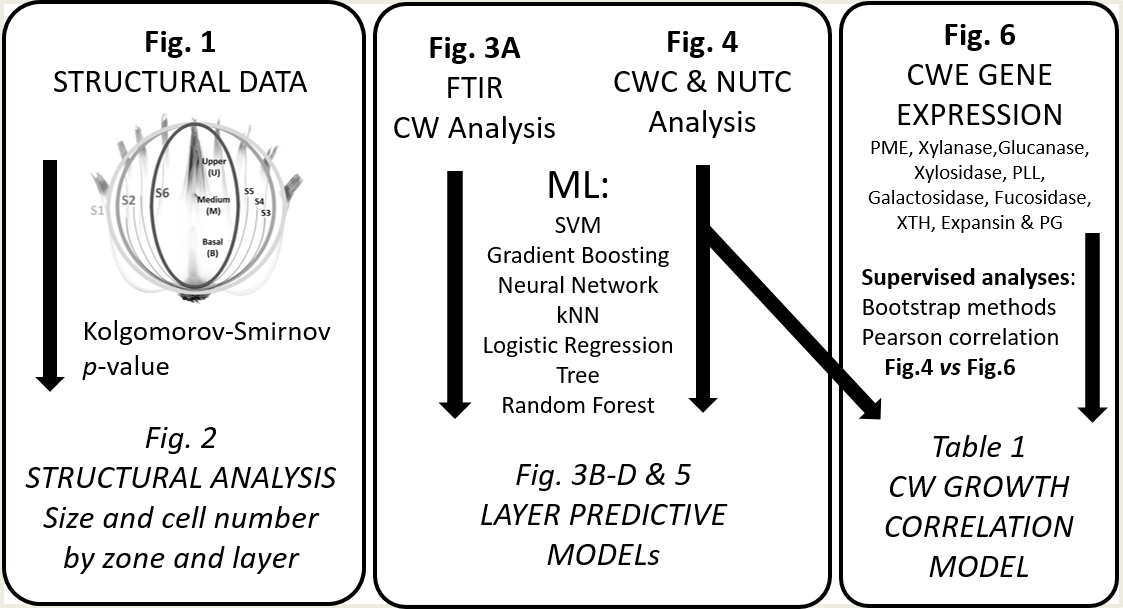

Supplement: Supplementary file 1 [file ijms-26-02946-s001.zip › Figure S1.png]

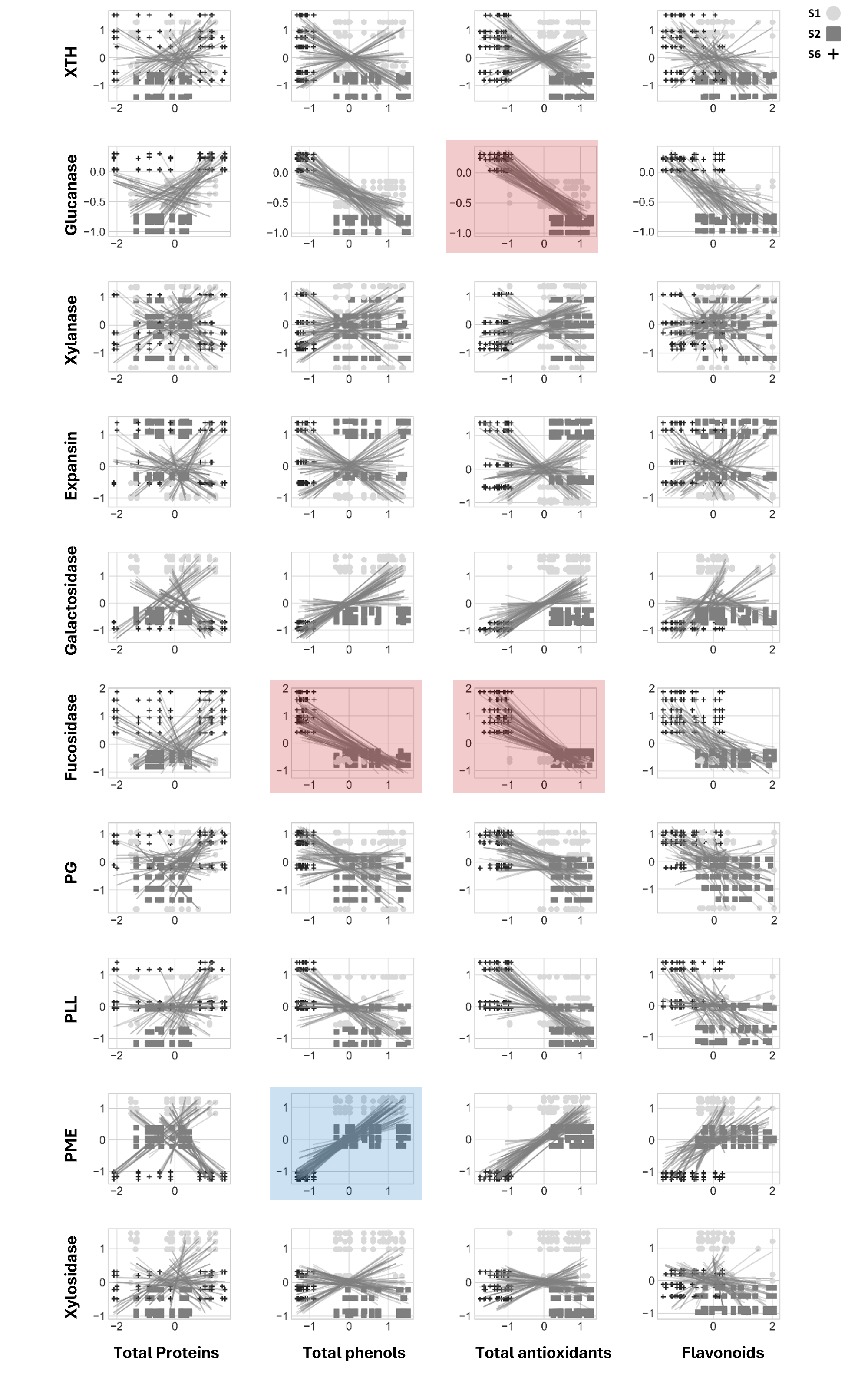

Supplement: Supplementary file 1 [file ijms-26-02946-s001.zip › Figure S2 _B_rev.png]

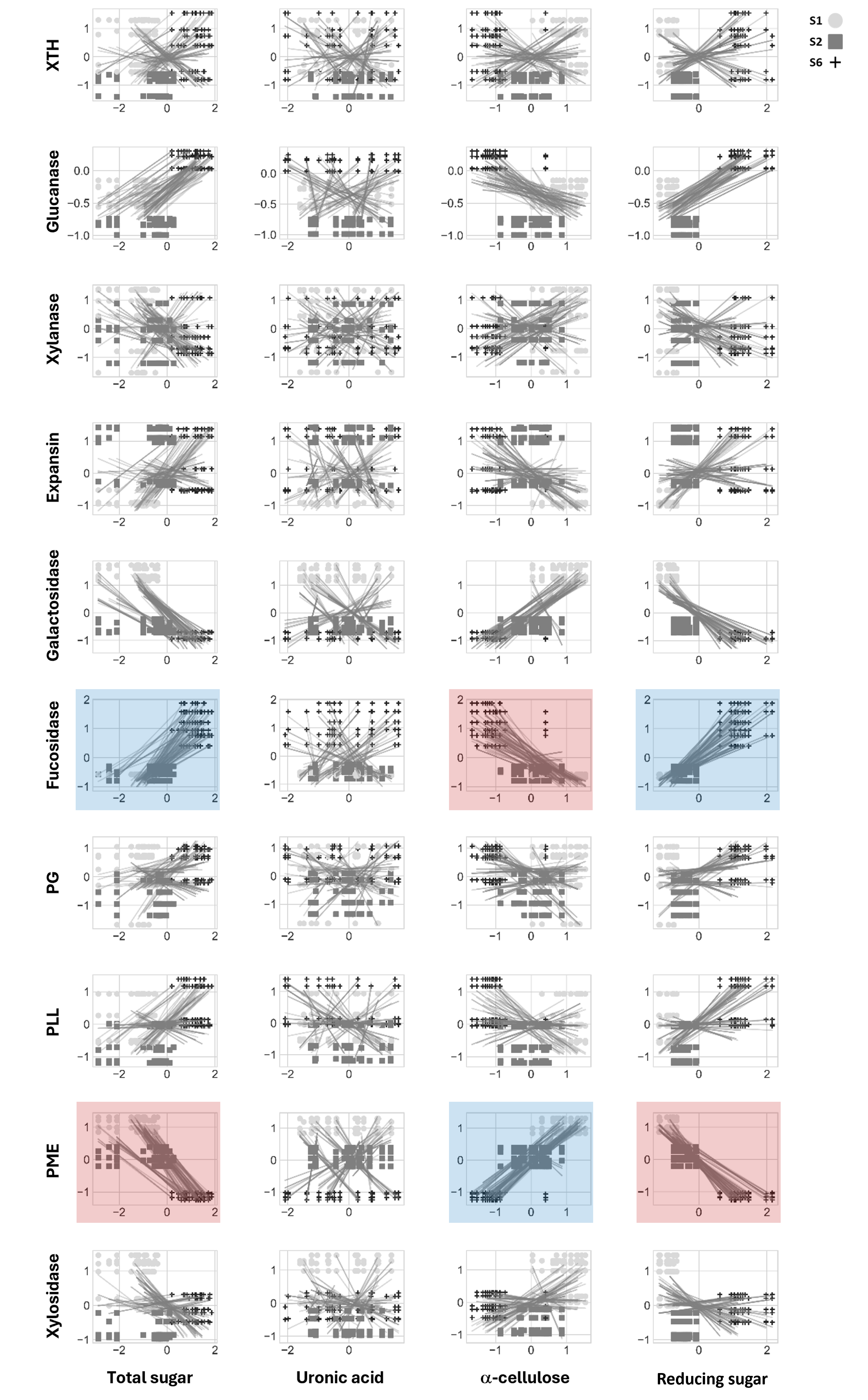

Supplement: Supplementary file 1 [file ijms-26-02946-s001.zip › Figure S2_A_rev2_20 03 2025.png]

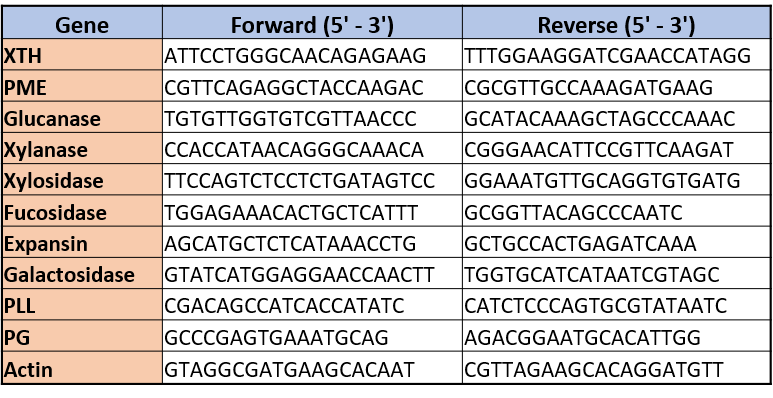

Supplement: Supplementary file 1 [file ijms-26-02946-s001.zip › Table S1.png]

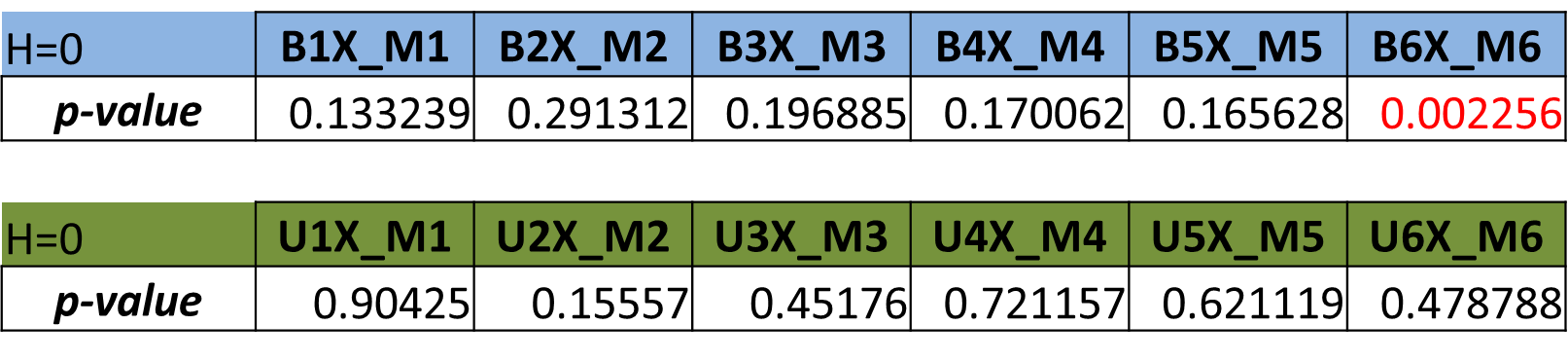

Supplement: Supplementary file 1 [file ijms-26-02946-s001.zip › Table S2_rev.png]

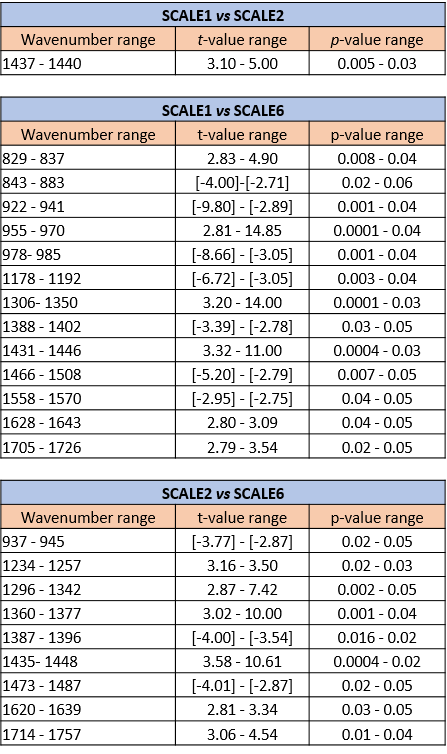

Supplement: Supplementary file 1 [file ijms-26-02946-s001.zip › Table S3.png]
